# Anomalous papillary muscle insertion into the mitral valve leaflet in hypertrophic obstructive cardiomyopathy: a lip nevus sign in echocardiography

**DOI:** 10.3389/fcvm.2023.1292142

**Published:** 2023-12-06

**Authors:** Jian Liu, Tong Tan, Peijian Wei, Jianrui Ma, Lishan Zhong, Hailong Qiu, Shengwen Wang, Jian Zhuang, Wei Zhu, Huiming Guo, Jimei Chen

**Affiliations:** ^1^ Guangdong Cardiovascular Institute, Guangdong Provincial People’s Hospital, Guangdong Academy of Medical Sciences, Guangzhou, Guangdong, China; ^2^ Guangdong Provincial Key Laboratory of South China Structural Heart Disease, Guangzhou, Guangdong, China; ^3^ Beijing Anzhen Hospital, Capital Medical University, Beijing Institute of Heart, Lung and Blood Vascular Diseases, Beijing, China; ^4^ Division of Adult Echocardiography, Guangdong Provincial People's Hospital (Guangdong Academy of Medical Sciences), Guangzhou, China

**Keywords:** anomalous papillary muscles, subvalvular malformation, hypertrophic obstructive cardiomyopathy, echocardiography, surgical outcomes, imaging modalities

## Abstract

**Background:**

Anomalous papillary muscle (APM) insertion into the mitral valve leaflet is rare but clinically important in hypertrophic obstructive cardiomyopathy (HOCM). In this study, we report the detection rate of APM insertion into the mitral valve using preoperative imaging modalities and the surgical outcomes of the patients.

**Methods:**

By retrospectively reviewing the clinical records of patients with HOCM who underwent surgical treatment by a single operation group at our center from January 2020 to June 2023, patients with APM insertion into the mitral valve leaﬂet were identified. Baseline data, image characteristics, and surgical outcomes were analyzed.

**Results:**

The incidence of APM insertion into the mitral valve leaﬂet was 5.1% (8/157). The insertion site was located at A3 in six cases, which was more common than at A2 (*n *=* *2). Preoperative echocardiography was used to identify two patients (25%) with APM insertion. We observed a particular echocardiographic feature for APM in HOCM patients, which was noted as a “lip nevus sign”, with a higher detection rate (62.5%). All patients successfully underwent septal myectomy with concomitant APM excision or mitral valve replacement via the transaortic (*n *=* *5) or transmitral (*n *=* *3) approach. The mean age was 49.0 ± 17.4 years and seven patients (87.5%) were female. Interventricular septum thickness (17.0 mm vs. 13.3 mm, *P *=* *0.012) and left ventricular outflow gradient (117.5 mmHg vs. 7.5 mmHg, *P *=* *0.012) were significantly decreased after surgery. Residual outflow obstruction, systolic anterior motion, and ≥3+ mitral regurgitation were negative. During the follow-up of 26.2 ± 12.2 months, there were no reported operations, adverse events, mitral regurgitation aggravations, recurrences of outflow obstruction, or instances of SAM.

**Conclusions:**

Papillary muscles inserted into the mitral valve leaﬂet are a subtype of subvalvular malformation in HOCM that requires surgical correction. The lip nevus sign on echocardiography is a characteristic of APM insertion in HOCM and may improve the preoperative detection rate. Adequate myectomy with anomalous papillary muscle excision has achieved good results in reducing the outflow gradient and eliminating mitral regurgitation, with good outcomes at short-to-intermediate follow-up.

## 1. Introduction

In hypertrophic obstructive cardiomyopathy (HOCM), left ventricular outflow tract (LVOT) obstruction is well established with respect to defined anatomical landmarks such as the hypertrophic septum. The mitral valve apparatus and subvalvular abnormalities are associated with systolic anterior motion (SAM) and related LVOT obstruction (1–3). Invasive septal reduction treatments, such as percutaneous alcohol septal ablation and surgical extended myectomy, are necessary for some symptomatic HOCM patients. However, the decision regarding the specific treatment approach is not always a one-size-fits-all situation, with imaging findings like echocardiography playing a major role in determining the most suitable course of action (4). For instance, HOCM patients with intrinsic mitral valve diseases or valve apparatus abnormalities may derive greater benefit from surgical treatment. Several studies have mentioned that attention should be paid to anomalous papillary muscles (APM) during the extended myectomy procedure (5, 6). Likewise, in the case of HOCM with APM, invasive surgery was the preferred treatment strategy due to the need to manage APM; however, this was based on the premise that APM was detected by preoperative echocardiography, a widely performed and accessible examination in the context of HOCM management. The insertion of APM into the mitral valve leaflet is a rare occurrence, attributed not only to its low incidence but also to the challenges associated with its detection. Because imaging detection of APM insertion into the mitral valve and the associated surgical outcomes have been reported infrequently, the aim of the study was to examine these aspects and provide new insights into the characteristics of APM as observed through echocardiography.

## 2. Methods

### 2.1. Study design and patient selection

This study is a retrospective cross-sectional study of consecutive patients with a diagnosis of HOCM seen in the Guangdong Cardiovascular Institute, Guangdong Provincial People's Hospital. A total of 157 patients with HOCM who underwent surgical correction by a single operation group at our center from January 2020 to June 2023 were included. The medical history, auxiliary inspection data, and surgical outcomes were collected from our inpatient electronic medical record system. We reviewed their preoperative echocardiography, cardiac magnetic resonance imaging (MRI), and computed tomography (CT) records to assess the presence of APM insertion into the mitral valve. The echocardiography was reviewed by two experienced cardiac sonographers, and two radiologists in the laboratory of artificial intelligence and 3D technology for cardiovascular diseases department were responsible for the MRI and CT records. Patients with low-quality imaging data were excluded. APM insertion into the mitral leaflet was confirmed by individual surgical records.

### 2.2. Surgical intervention

**Figure 1:**
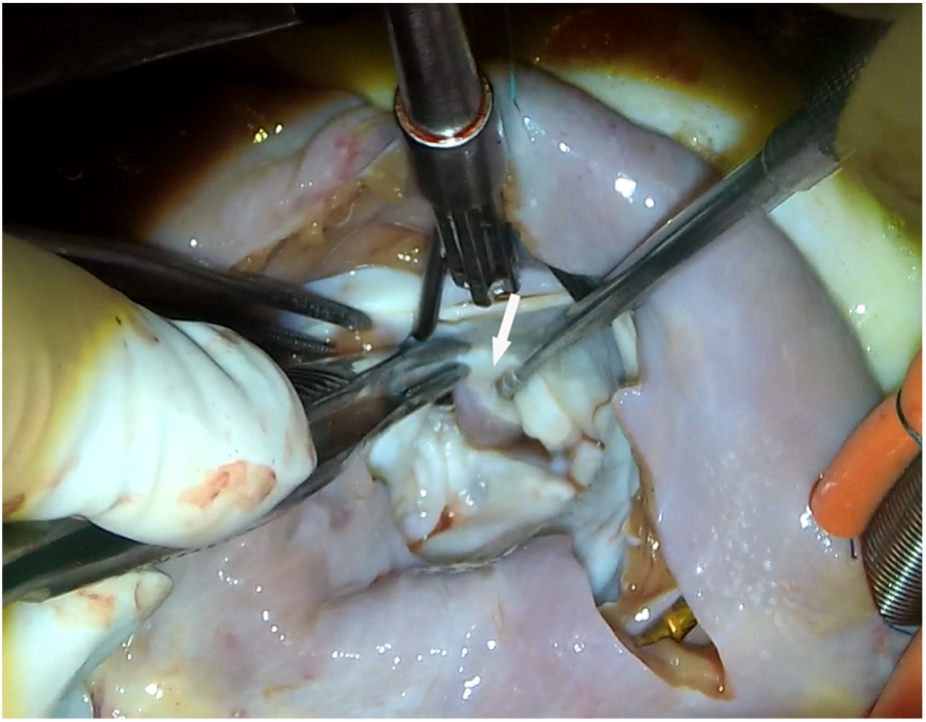
View of anomalous papillary muscles (arrow) directly inserted into the anterior mitral valve.

Symptomatic patients with HOCM who were tolerant to medication and had ≥50 mmHg of left ventricular outflow tract gradient (LVOTG) received surgical treatment as recommended by the management guideline for HOCM (7). Extended myectomy, via the transaortic or transmitral approach, was performed as described previously (8). Surgical strategies for mitral valves and APMs include mitral valve replacement, papillary muscle dissociation and excision, and other mitral repair techniques, as needed. When the subvalvular APM (Figure 1) is identified, its insertion site and surrounding chordae tendineae should be explored using a retractor. In case of valve injury, the APM insertion into the body of the leaflet was cut approximately 1 cm from the leaflet. The hypertrophic papillary muscles were partially sliced to palliate the narrow space within the left ventricle.

### 2.3. Surgical outcome assessment

The surgical outcome data included the success rate of surgery, major adverse cardiovascular events, and echocardiographic parameters of HOCM. Major adverse cardiovascular events include death, reoperation, severe arrhythmias (such as new-onset atrial fibrillation), myocardial infarction, stroke, progressive heart failure, and sudden death. Echocardiography before discharge and during the follow-up period was used to analyze the clinicopathological indices of HOCM, including interventricular septum (IVS) thickness, LVOTG, degree of mitral regurgitation (MR), SAM, and other abnormalities within the cardiac cavity.

### 2.4. Statistical analysis

Data are expressed as *N* (%), mean ± standard deviation, or median (interquartile range) where appropriate. Statistical analyses were conducted using SPSS software. Differences in continuous variables between the groups were analyzed using Student's *t*-test or a Mann–Whitney *U*-test. Fisher's exact test was performed to compare the counting data between groups.

## 3. Results

Of the 157 patients in our series, eight patients with HOCM (1 male and 7 females) that had APM insertion into the mitral valve leaﬂet were identified by surgical records. The baseline characteristics are summarized in Table 1. The mean age was 49.0 ± 17.4 years. The most common clinical symptom was chest distress (62.5%); one patient had a history of syncope, and one asymptomatic patient with HOCM was found during the annual physical examination. Six (75.0%) patients were on β-blocker therapy before hospitalization. All APMs were anterolateral papillary muscles. The insertion sites were located at A3 (75%) and A2 (25%). The median IVS was 17.0 (16.0, 23.3) mm. Compared with the IVS [20.0 (18.0, 24.0) mm] of patients with HOCM without APM in this cohort (Table 2), the IVS was thinner, but the difference was not statistically significant (*P* = 0.595). However, the median LVOTG was 117.5 (82.3, 136.8) mmHg, which showed a significant increase compared with the non-APM group [85.0 (67.5, 99.8) mmHg, *P *=* *0.034]. Two patients (25.0%) had midventricular obstruction. Hypertrophic papillary muscles were found in two patients (25.0%) and chordal rupture was found in one patient (12.5%). Mild to severe SAM was observed in seven patients (87.5%), contributing to a ≥3+ degree of MR in five patients (62.5%).

**Table 1:** Baseline characteristics of hypertrophic obstructive cardiomyopathy patients with anomalous papillary muscles inserting into the mitral valve.

Characteristic	*N *= 8
Female	7 (87.5%)
Age, years	49.0 ± 17.4
BMI, kg/m^2^	22.0 ± 0.9
Hypertension	2 (25.0%)
History of PCI	1 (12.5%)
Stroke history	1 (12.5%)
Family history of cardiomyopathy	0
NYHA class≥III	7 (87.5%)
EuroSCORE II, %	1.7 ± 1.0

BMI, body mass index; PCI, percutaneous coronary intervention; NYHA, New York Heart Association.

**Table 2:** Echocardiographic parameters of HOCM patients with and without anomalous papillary muscles.

Variable	APM (*n *=* *8)	Non-APM (*n *=* *149)	*P*-value
IVS, mm	17.0 (16.0, 23.3)	20.0 (18.0, 24.0)	0.206
LVOTG, mmHg	117.5 (82.3, 136.8)	85.0 (67.5, 99.8)	0.034
SAM	7 (87.5%)	130 (87.2%)	1.000
MR≥3+	5 (62.5%)	116 (77.9%)	0.385
EF, %	66.0 ± 2.9	67.8 ± 4.2	0.227
LAD, mm	41.1 ± 3.4	43.0 ± 7.3	0.462
LVDD, mm	42.6 ± 4.8	41.6 ± 5.0	0.589

IVS, interventricular septum; LVOTG, left ventricular outflow tract gradient; SAM, systolic anterior motion; MR, mitral regurgitation; EF, ejection fraction; LAD, left atrial diameter; LVDD, left ventricular diastolic diameter.

**Figure 2:**
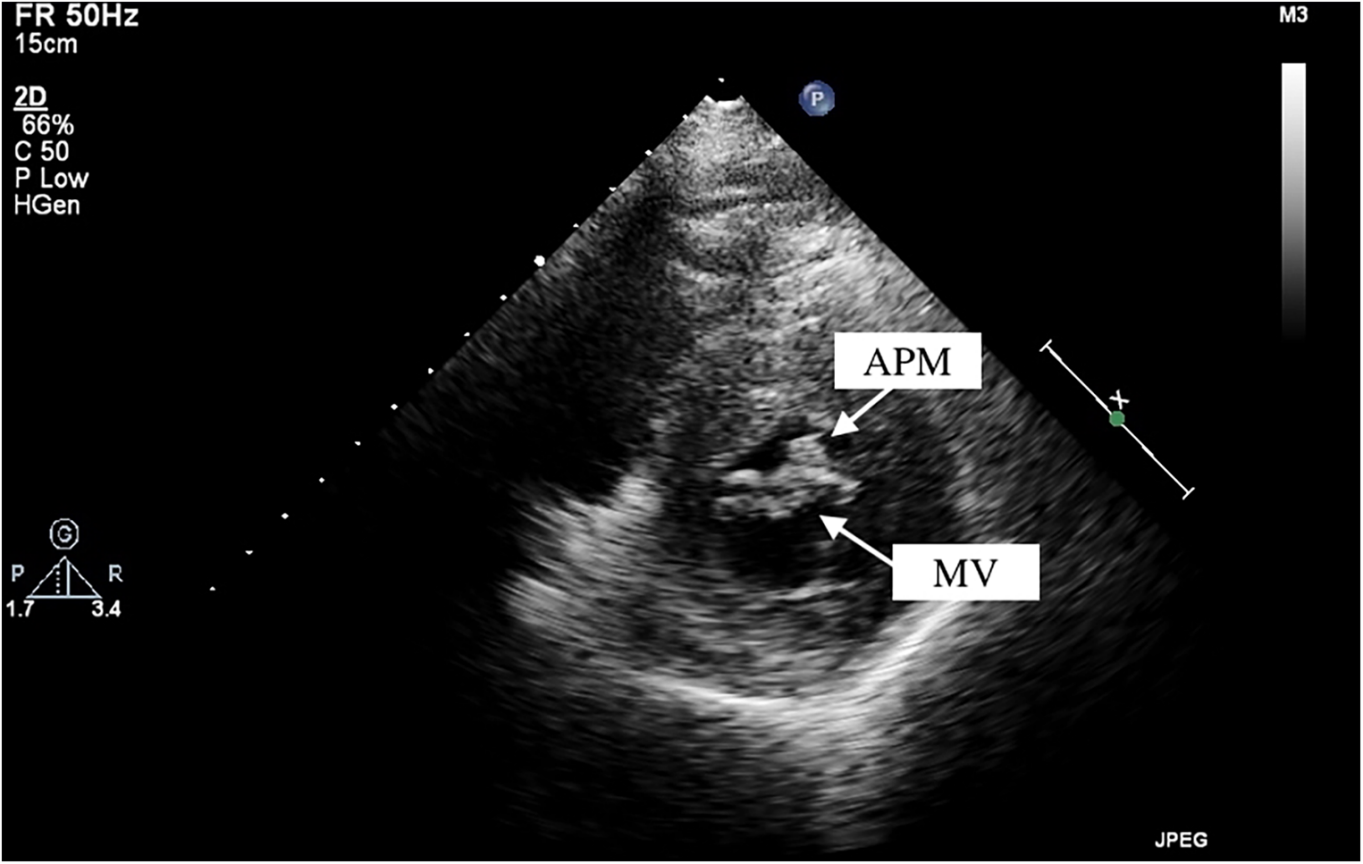
Anomalous papillary muscle (upper arrow) insertion into the mitral valve (lower arrow) visualized by transthoracic echocardiography. APM, anomalous papillary muscle; MV, mitral valve.

Among these patients with HOCM, two cases of APM insertion were preoperatively detected using transthoracic echocardiography, with a sensitivity of 25%. CT was performed in seven patients, and MRI was available for six patients. However, only two and one cases of APM insertion were operatively detected, leading to sensitivities of 28.6% and 16.7% for CT and MRI, respectively. By reviewing the echocardiographic images, we observed a particular feature of APM insertion: the APM adhering to the leaflet looked like a lip nevus in the horizontal cross-section (Figure 2 and Supplementary Video S1). By contrast, Supplementary Video S2 shows the echocardiography findings for HOCM without APM insertion. The “lip nevus sign” was found in 62.5% (5/8) of patients.

The extended myectomy and APM resection procedures were successfully performed. In this series, the two eldest patients (a 62-year-old and 69-year-old) and one patient with midventricular obstruction underwent myectomy via a minimally invasive transmitral approach; they had concomitant mitral valve replacement. The other five patients underwent thoracotomy; one patient required coronary artery bypass grafting, one underwent ventricular aneurysmectomy, and one underwent posterior band annuloplasty with artificial chord implantation. The mean cardiopulmonary bypass time was 144.4 ± 31.9 min, and the aortic cross-clamp time was 82.9 ± 21.7 min. Postoperative ICU stay length was 2.6 (1.8, 4.5) days. Except for one case of persistent pleural effusion, there were no instances of death, reoperation, new-onset atrial fibrillation, permanent pacemaker implantation, or other adverse cardiovascular events before discharge. The postoperative echocardiography showed that IVS [17.0 (16.0, 23.3) mm vs. 13.3 (11.3, 14.0) mm, *P *=* *0.012) and LVOTG [117.5 (82.3, 136.8) mmHg vs. 7.5 (5.0, 15.5) mmHg, *P *=* *0.012) were significantly decreased. One patient had a residual mid-ventricular obstruction (55 mmHg) due to asymmetric mid-septal hypertrophy, which was difficult to resect via the transaortic approach. Postoperative ≥3+ MR and SAM were negative. Ejection fraction, left atrial diameter, and left ventricular diastolic diameter were 61.3% ± 7.9%, 35.9 ± 4.3 mm, and 38.9 ± 2.6 mm, respectively. The cutoff date for follow-up was 15 July 2023. All patients survived, without any reoperation or adverse events reported during the 26.2 ± 12.2 months follow-up period. Echocardiographic parameters remained consistent with the postoperative measurements—the LVOTG was 3.0 (1.0, 13.6) mmHg; three patients had no MR, while the other five patients remained 2+ MR; SAM disappeared in all cases. Left ventricular diastolic diameter exhibited a reduction due to the improvement in MR compared with the preoperative data (37.5 ± 3.2 mm vs. 42.6 ± 4.8 mm, *P *=* *0.022).

## 4. Discussion

HOCM with APM insertion is a rare phenotype that has mostly been described in case reports or series. The reported incidence of APM insertion ranges from 4% to 13% (9–11). Most studies on APM in HOCM have reported it to be a significant pathological component of dynamic obstruction (12–15). Although the papillary muscles and chordae tendineae have anatomical variations in origin sites, sizes, and number of bellies, the tendinous cords usually interconnect the mitral valve leaflet and papillary muscles; hence, such interposition affects the opening and closing of the mitral valve. Congenital failure of chordal development at 11–13 weeks of gestation leads to APM insertion into the mitral valve leaflets (16, 17). The APM (especially the anterolateral one) directly inserted into the mitral valve leaflet provides a rigid structure that occupies a cavity between the ventricular wall and the left ventricular outflow tract; therefore, it aggravates the gradient of the outflow tract or mid-ventricle during the systolic phase. In a severe case (18), a long and profoundly hypertrophied APM divides the ventricular cavity into two chambers. In addition to the space-occupying effect, abnormal fluid dynamics along the APM may cause shear stress and trigger fibrotic tissue growth in the septal wall, causing dynamic obstruction (19, 20). Carvalho et al. (11) reported the largest sample size (*N* = 73) for APM insertion and categorized them into three types depending on the insertion site. Specifically, APM inserting directly into the body of the anterior leaflet, the free margin, or both, were classified as type I, III, and II, respectively. This classification system, along with the findings of the study, underscores the significance of type I and II APM in addition to myocardial hypertrophy, all of which collectively influence treatment decisions (21, 22). Along with the APM insertion into the leaflet, the coexistence of other abnormalities, including accessory papillary muscles (most common), fusion to the ventricle wall/septum, hypertrophic papillary muscles, chordae slack, biﬁd papillary muscles, and APM anterior displacement, was found. These combinations also contribute to a dynamic obstruction, which requires concomitant surgical palliation.

SAM is a classical pathophysiological characteristic of HOCM, in which the anterior mitral valve leaflet is dragged into the outflow tract and comes into contact with the IVS. The factors contributing to SAM have been well studied and are divided into three categories: structural, geometric, and kinetic (23, 24). A bulging septum is the most common cause of SAM; however, APM, a subvalvular mitral valve apparatus anomaly, is another structural factor that independently promotes SAM. A fixed-length APM that is directly inserted into the anterior mitral valve leaflet tethers the leaflet itself; thus, the leaflet is anteriorly displaced toward the outflow tract, and the distance between the septum and the coaptation point is shortened. These anomalies finally result in SAM and MR, which even occurs in non-HOCM patients (25, 26).

**Figure 3:**
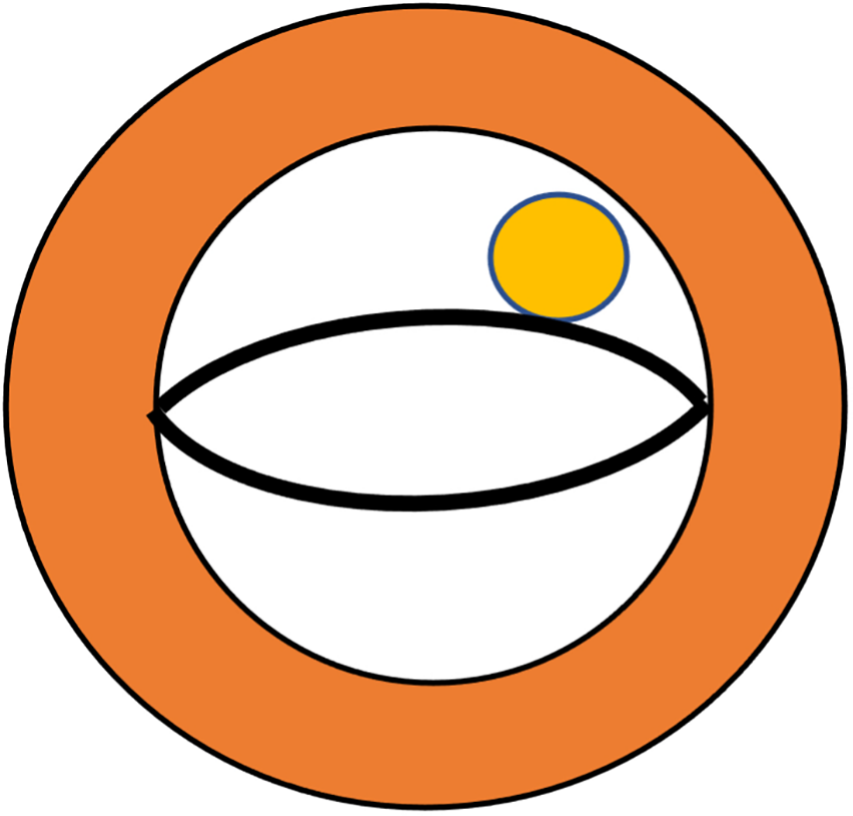
Schematic demonstration of the lip nevus sign (yellow) in echocardiography to define anomalous papillary muscles directly inserted into the mitral valve leaflet.

Our primary finding is the “lip nevus sign” (Figure 3) in 2D-echocardiography, which has a high detection rate in our study, and could be used to identify the APM directly inserting into the mitral valve leaflet. Diagnosing APM demands a degree of expertise and knowledge about it. The diagnostic challenge is exacerbated by variations in the acoustic window, making it challenging to differentiate APM from a thickened free edge of the leaflet, hypertrophic chordae tendineae, or secondary papillary muscles. Consequently, there is a compelling need for a distinctive visual feature in image examinations. In the short axis of the mitral valve, the anterior and posterior leaflets were similar to those of human lips. A circular muscular structure that tightly adheres to the leaflet constitutes the “lip nevus sign” as a characteristic ultrasound finding. In addition, in the apical four- or three-chamber view, a long muscular structure overriding the body of the mitral valve leaflet and the surrounding papillary muscles indicates APM insertion. Sonographers should regard APM detection as a regular step in the evaluation of HOCM. It is essential to observe the location, morphology, hypertrophy, and movement of the papillary muscles within the most lucid visual field attainable. The presence of restricted mitral leaflet mobility should prompt suspicion of APM as the aberrant attachment of the leaflet to a fixed structure (e.g., the ventricle wall) may be the underlying etiology. This will lead the examiner to perform detailed examination, even multimodality imaging (27). Furthermore, APM can be considered when the identified structural anomalies could not explain all the pathophysiological manifestations. For example, in the scenario of mild degree of septal hypertrophy but severe MR without mitral valve diseases, APM might be the factor exacerbating MR. Likewise, the dynamic obstruction mechanisms through APM insertion is most commonly and independently derived from its space-occupying effect instead of SAM (28). In our study cohort, HOCM patients with APM showed a significantly higher LVOT gradient than those without APM, suggesting that this could also serve as a diagnostic clue for APM. In a word, when cardiac ultrasound typically finds a mismatch between a large outflow gradient and a less serious SAM or septal hypertrophy, APM insertion should be considered. With these technical advantages, more APM insertions may be detected by echocardiography. In this study, we also found that APM insertion occurred frequently in the anterolateral group, with the insertion site at A2 or A3. Whether the precise insertion site has significant impacts on the dynamic change in HOCM remains an open question, necessitating further studies with a larger cohort.

In addition to echocardiography, MRI and CT are the main imaging modalities used for the diagnosis of APM insertion, which provide more details of the papillary muscles and mitral valve with higher resolution and multiplanar capabilities (13, 29, 30). A continuous muscular-like density structure between the mitral leaflet and ventricular free wall is a sign of APM insertion in three-dimensional images. However, this has usually been overlooked, as was found in our study. As previously described (31), the three-dimensional printing technique is an adjunct to common imaging examinations and helps surgeons achieve an intuitive visualization of APM insertion. It can not only identify the APM insertion preoperatively but also assist in surgical planning by providing more details such as the diameter of the APM and a simulated view of the surgical approach. Nevertheless, given the comparable sensitivity and popularity of echocardiography, these three-dimensional imaging modalities are not the principal examinations for all patients with HCOM (7).

APM insertion into the mitral valve leaflet usually requires excision to relieve the obstruction, unless it is inserted into the free edge of the mitral valve (32). In our experience, cutting off the APM does not affect valve movement. Both extended myectomy and APM dissociation from the mitral valve are necessary for the treatment of HOCM. Leaving any anomalies increases the risk of residual obstruction. Therefore, we performed the extended myectomy procedure as usual and sacrificed all the inserted APM. No secondary prolapse occurred, with normal papillary muscles and chordae tendineae preserved. Some studies have demonstrated feasible outcomes of APM insertion treated using mitral repair techniques (6, 33). Except in intrinsic mitral valve disease, the valve leaflet itself is less likely to cause obstruction or SAM. Our study provides additional clinical evidence supporting this finding. Further studies are recommended to determine the long-term effects of these two surgical tactics.

There are some limitations to this study that warrant consideration. First, the overall sample size of this cohort was small due to the low incidence rate of APM. Although the lip nevus sign is proven to be a practical and visualized feature in echocardiography, further studies with large sample sizes encompassing patients with and without HOCM are clearly needed to verify its prevalence, as well as the sensitivity and specificity. Second, the preoperative imaging examinations were performed and reported by doctors with different seniority; however, detection of APM lacks a standardized benchmark in imaging diagnostics instead of intraoperative exploration. Consequently, the reliability of such diagnoses relies on the experience of cardiac sonographers, which may introduce a bias in detection rates. Third, given that only eight patients were identified as having APM in this study, and the morphology and original sites displayed similarities, this helps us propose the concept of the lip nevus sign; however, it is hard to categorize them into subtypes. The surgical treatment for APM in this study involved a resection strategy only. Alternative treatments, such as shaving or mobilization APM, may be more appropriate depending on the subtypes; therefore, we are unable to conclude what the optimal surgical strategy may be for each type of APM at this stage.

## 5. Conclusion

Papillary muscles inserted into the mitral valve leaﬂet are a subtype of subvalvular malformation in HOCM that requires surgical correction. The lip nevus sign on echocardiography is a characteristic of APM insertion and may improve the preoperative detection rate. Adequate myectomy with APM excision has achieved good results in reducing the outflow gradient and eliminating mitral regurgitation, with good outcomes at short-to-intermediate follow-up.

## Data Availability

The original contributions presented in the study are included in the article/**Supplementary Material**, further inquiries can be directed to the corresponding authors.
