# Corrigendum: Anomalous papillary muscle insertion into the mitral valve leaflet in hypertrophic obstructive cardiomyopathy: a lip nevus sign in echocardiography

**DOI:** 10.3389/fcvm.2024.1395480

**Published:** 2024-03-21

**Authors:** Jian Liu, Tong Tan, Peijian Wei, Jianrui Ma, Lishan Zhong, Hailong Qiu, Shengwen Wang, Jian Zhuang, Wei Zhu, Huiming Guo, Jimei Chen

**Affiliations:** ^1^ Guangdong Cardiovascular Institute, Guangdong Provincial People’s Hospital, Guangdong Academy of Medical Sciences, Guangzhou, Guangdong, China; ^2^ Guangdong Provincial Key Laboratory of South China Structural Heart Disease, Guangzhou, Guangdong, China; ^3^ Beijing Anzhen Hospital, Capital Medical University, Beijing Institute of Heart, Lung and Blood Vascular Diseases, Beijing, China; ^4^ Division of Adult Echocardiography, Guangdong Provincial People’s Hospital (Guangdong Academy of Medical Sciences), Guangzhou, China

**Keywords:** anomalous papillary muscles, subvalvular malformation, hypertrophic obstructive cardiomyopathy, echocardiography, surgical outcomes, imaging modalities

A Corrigendum on Anomalous papillary muscle insertion into the mitral valve leaﬂet in hypertrophic obstructive cardiomyopathy: a lip nevus sign in echocardiography By Liu J, Tan T, Wei P, Ma J, Zhong L, Qiu H, Wang S, Zhuang J, Zhu W, Guo H and Chen J (2023). Front. Cardiovasc. Med. 10:1292142. doi: 10.3389/fcvm.2023.1292142

In the published article, there was an error in affiliation 1. Instead of “Department of Cardiovascular Surgery, Guangdong Cardiovascular Institute, Guangdong Provincial People's Hospital (Guangdong Academy of Medical Sciences), Southern Medical University, Guangzhou, Guangdong, China”, it should be “Guangdong Cardiovascular Institute, Guangdong Provincial People's Hospital, Guangdong Academy of Medical Sciences, Guangzhou, Guangdong, China,”.

The authors apologize for this error and state that this does not change the scientific conclusions of the article in any way. The original article has been updated.

